# Machine Learning and the Use of Spectroscopy for Adulteration Detection in Turmeric Powder

**DOI:** 10.3390/molecules31101774

**Published:** 2026-05-21

**Authors:** Asma Kisalaei, Vali Rasooli Sharabiani, Ahmad Banakar, Ebrahim Taghinezhad, Mariusz Szymanek, Agata Dziwulska-Hunek

**Affiliations:** 1 Department of Biosystems Engineering, Faculty of Agriculture and Natural Resources, University of Mohaghegh Ardabili, Ardabil 56199-11367, Iran; a.kisalaei@gmail.com (A.K.); vrasooli@uma.ac.ir (V.R.S.); 2 Biosystems Engineering Department, Tarbiat Modares University, Tehran 14117-13116, Iran; 3 Department of Agricultural, Forest and Transport Machinery, University of Life Sciences in Lublin, Głęboka 28, 20-612 Lublin, Poland; 4 Department of Biophysics, University of Life Sciences in Lublin, 20-950 Lublin, Poland

**Keywords:** machine learning, turmeric, feature selection methods, spectroscopy

## Abstract

This research aimed to develop a rapid, non-destructive, and accurate method for detecting adulteration in turmeric using Visible–Near-Infrared (UV/Vis and NIR) spectroscopy combined with machine learning algorithms. Spectral data from the samples were collected and analyzed in two ranges: 170–870 nm (UV/Vis) and 900–2170 nm (NIR). Four supervised learning algorithms, including Support Vector Machine (SVM), Linear Discriminant Analysis (LDA), the Multilayer Perceptron (MLP) neural network, and Decision Tree, were evaluated for modeling. To quantitatively assess model performance, we employed not only the accuracy metric but also complementary performance indicators including precision, recall, and the F1-score to provide a more comprehensive evaluation of classification effectiveness. The models developed in the 900–2170 nm spectral range demonstrated highly significant performance, with most models achieving 100% accuracy on the independent test set. To reduce data dimensionality and enhance computational efficiency, a hybrid feature selection method combining SVM with five algorithms—League Championship Algorithm (LCA), Genetic Algorithm (GA), Particle Swarm Optimization (PSO), Ant Colony Optimization (ACO), and Imperialist Competitive Algorithm (ICA)—was employed. Upon evaluation of each method, the SVM-LCA was selected as the optimal feature selection technique. This algorithm successfully extracted the most effective wavelengths with the highest correlation and lowest error, which maintained or improved the accuracy of the classification models. This study confirms the high potential of UV/Vis and NIR spectroscopy as rapid, non-destructive, and precise tools for detecting adulteration in turmeric. The findings can pave the way for the development of intelligent quality control systems in the food and pharmaceutical industries, playing a crucial role in ensuring consumer health and safety.

## 1. Introduction

Turmeric (*Curcuma longa*) is one of the most widely used medicinal plants and spices, holding a special place in the food, pharmaceutical, and traditional medicine industries due to its medicinal properties and bioactive compounds, particularly curcumin. This plant belongs to the ginger family (Zingiberaceae) and is extensively cultivated in the tropical regions of South and Southwest Asia [1]. Curcumin, the principal bioactive compound in turmeric, is a yellow polyphenol possessing antioxidant, anti-inflammatory, anticancer, and antibacterial properties. This compound plays a significant role in the treatment of diseases such as diabetes, cardiovascular diseases, neurological disorders, and autoimmune conditions [1]. Studies have shown that curcumin can inhibit the growth of cancer cells and prevent metastasis. Furthermore, this compound is effective in the prevention of gastric and intestinal cancers [2]. Given its high demand and relatively high price, this product is often a target for economic adulteration. Adulteration practices such as the addition of inexpensive powders, artificial colors like Metanil yellow and Sudan dyes, or even more hazardous compounds such as lead chromate have been reported, which can threaten consumer health [3]. Artificial colors like Metanil yellow and Sudan dyes used in adulterated turmeric may cause skin irritation, allergies, and even cancer. Moreover, the use of lead chromate as a colorant is associated with severe toxic effects, damage to the nervous system and kidneys, and the induction of genetic mutations. The addition of low-cost materials like starch or flour, while reducing the quality and nutritional value of turmeric, can also lead to digestive problems. Therefore, the consumption of adulterated turmeric not only diminishes the product’s quality but also poses serious health risks.

Common methods for detecting adulteration in turmeric primarily involve sophisticated analytical chemistry techniques like High-Performance Liquid Chromatography (HPLC), Gas Chromatography (GC), Atomic Absorption Spectroscopy (AAS), and Mass Spectrometry (MS). These methods are highly capable of identifying and quantifying adulterants such as synthetic dyes, heavy metals, or chemical impurities. For example, HPLC can accurately detect the presence of synthetic dyes like Metanil Yellow or Sudan in a sample [4], while AAS is very effective for measuring heavy metals like lead and chromium. However, these techniques typically require complex sample preparation, expensive chemicals, specialized laboratory equipment, and trained personnel. They are also destructive, meaning the sample cannot be used after testing. Additionally, these tests are time-consuming and not suitable for high-scale industrial applications. In contrast, Near-Infrared (NIR) Spectroscopy is recognized as a modern, non-destructive, and rapid method for detecting food adulteration, including in turmeric. This technique is based on the absorption of light in the near-infrared region (700–2500 nm) and the analysis of the spectral characteristics of the sample’s chemical compounds. Without the need for complex preparation or chemicals, NIR spectroscopy can provide precise information about a sample’s chemical composition in a very short time. This technology becomes particularly powerful when combined with chemometric methods and machine learning algorithms such as Principal Component Analysis (PCA), Partial Least Squares Regression (PLSR), and Support Vector Machine (SVM). These algorithms are capable of identifying hidden patterns in spectral data and can accurately differentiate between authentic and adulterated samples [5]. The combination of spectral data from NIR with multivariate analysis and machine learning algorithms has shown a high capability to detect subtle chemical changes and identify hidden patterns in contaminated samples [6]. This combination not only allows for the classification of adulterated and authentic samples but can also predict the approximate level of impurities.

Due to the importance of rapid, accurate, and non-destructive detection of adulteration in spices, particularly turmeric, spectroscopic methods—especially Near-Infrared (NIR) spectroscopy—have gained increasing attention from researchers in recent years. Notable studies include the use of Vis-NIR spectroscopy combined with machine learning to identify impurities in turmeric powder and predict the level of adulteration with an error of less than 15% [7]. Time-of-Flight Secondary Ion Mass Spectrometry (TOF-SIMS) has been employed for the authenticity analysis of saffron, using molecular analysis to detect impurities such as safflower and turmeric [8]. The chemical impurity Metanil Yellow has been identified in turmeric powder using NIR spectroscopy and chemometric methods like Principal Component Regression and PLSR [9].

Further research has reviewed various types of adulterants and impurities in spices and medicinal plants, highlighting rapid and sensitive methods, including spectroscopy and image analysis, for their detection [10]. Economic adulteration in organic spices has been detected using NIR spectroscopy and chemometric modeling, which showed high accuracy in predicting the percentage of impurities [11]. The combination of Convolutional Neural Networks (CNNs) and chemometrics has been used for analyzing NIR spectra and RGB images to identify and track turmeric impurities with high precision and speed [12]. The use of Raman and Mid-Infrared spectroscopy along with deep learning (1D CNN) has enabled the accurate and rapid detection of impurities in powdered turmeric samples [13].

Additionally, Near-Infrared (NIR) spectroscopy combined with chemometric methods like PCA and LDA has successfully differentiated various turmeric brands [14]. The identification and quantification of two chemical impurities (Sudan Red and Metanil Yellow) in turmeric powder have been achieved using NIR and chemometric models following various spectral data preprocessing steps [15]. The rapid detection of adulteration in ginger powder has been demonstrated using FT-NIR and machine learning algorithms such as Random Forest and Gradient Boosting [16]. The potential of Energy-Dispersive X-ray Fluorescence (ED-XRF) spectroscopy for identifying mineral impurities in turmeric, paprika, and oregano has also been evaluated [17]. Starch adulteration in turmeric powder has been identified using FT-NIR and multivariate regression models [18], and the adulteration of saffron has been assessed using Diffuse Reflectance Infrared Fourier Transform Spectroscopy (DRIFTS) and chemometric methods to identify and quantify plant-based impurities [19].

Previous studies have demonstrated the efficacy of spectroscopic methods for classifying and identifying adulterated spices. However, these studies have typically focused on a limited number of adulterants and rarely addressed more complex, multi-adulterant conditions. Consequently, this study developed a non-destructive and accurate model for detecting adulterated turmeric by combining spectroscopy with advanced machine learning algorithms. A key distinction of this research from previous work is the use of a larger and more diverse set of adulterants introduced into the turmeric samples, thereby creating more realistic and complex challenges for impurity detection. By applying advanced machine learning techniques to spectroscopic data, the aim is to present an efficient and reliable model for detecting various types of adulteration with high accuracy and speed.

## 2. Results and Discussion

### 2.1. Analysis of Reflectance Spectra in the Visible-Shortwave Near-Infrared Region (430–870 nm)

**Figure 1:**
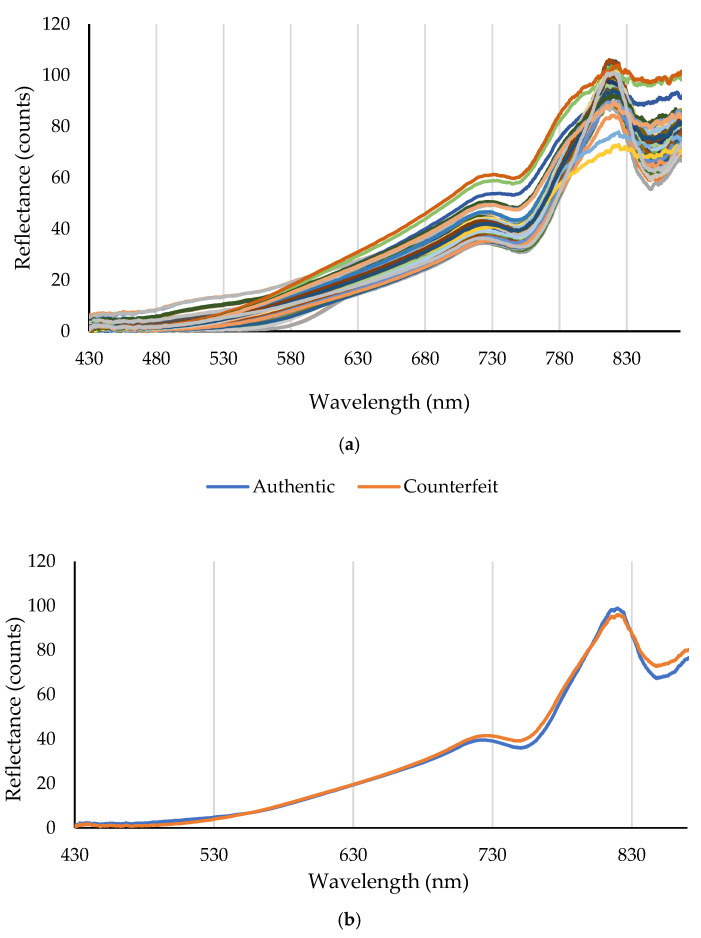
Spectral data graphs in the 430–870 nm range. (**a**) is the total spectral data, and (**b**) is the mean spectral data for authentic and counterfeit turmeric samples.

Figure 1a shows the distribution of reflectance spectra for all authentic and adulterated turmeric samples in the 430–870 nm range. As observed, the spectral patterns exhibit significant variations, reflecting compositional and structural differences among the samples due to the addition of various adulterants at different concentrations.

Figure 1b presents the average reflectance spectra of the authentic and adulterated samples. The spectrum of authentic turmeric shows low reflectance (high absorption) in the 430–530 nm region, primarily due to the presence of curcuminoid pigments, especially curcumin, which has a maximum absorption around 420–430 nm [20]. With increasing wavelength, the reflectance gradually rises, and after a gentle slope in the 530–680 nm range, it increases sharply from around 730 nm, reaching a distinct peak at approximately 810–820 nm. A small secondary feature is also visible as a peak around 850–860 nm.

The average spectrum of the adulterated samples, while retaining the overall pattern of authentic turmeric, shows significant changes in reflectance intensity. The most prominent change is the increase in reflectance (decrease in absorption) in the 530–780 nm region. This could be due to the dilution of curcuminoid concentration by the presence of bulking agents such as corn powder, talc, or fruit peel powder, which leads to a reduction in the intensity of the yellow color and a decrease in light absorption within this spectral range. Additionally, the reflectance peak at approximately 810–820 nm has a lower height in the adulterated samples compared to the authentic ones. A more detailed analysis of the individual spectra (Figure 1a) could enable the identification of potential artificial colors like Tartrazine and Sunset Yellow through new absorption peaks or spectral shape changes, although these effects may be less visible in the average adulterated spectrum due to the dispersion in the type and amount of adulteration.

### 2.2. Near-Infrared Reflectance Spectroscopy (900–2170 nm)

**Figure 2:**
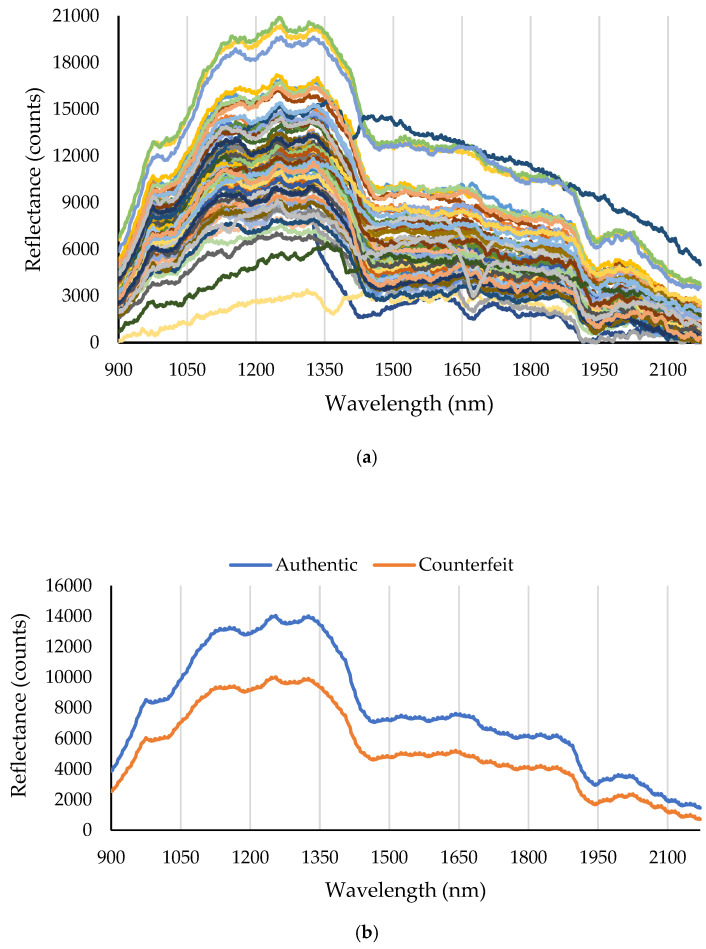
The spectral data in the 900–2170 nm range. (**a**) The complete spectral dataset. (**b**) The average spectral data of authentic and counterfeit turmeric samples.

Figure 2a illustrates the scatter plot of the near-infrared (NIR) reflectance spectra for all samples, clearly showing structural and compositional variations resulting from adulteration. NIR spectroscopy is sensitive to molecular vibrations of O–H, C-H, and N-H bonds, providing key information about the organic composition and moisture content of the samples.

Figure 2b presents the average reflectance spectra of genuine and adulterated turmeric samples. The spectra of genuine turmeric samples possess distinct features, including a peak around 970–980 nm, which is attributed to the second overtone of O–H bonds from water or hydroxyl groups present in starch and cellulose [21]. Additionally, a decrease in reflectance near 1200 nm indicates the presence of the second overtone of C–H bonds [22]. A high reflectance region between 1300 and 1350 nm and two deep absorption valleys—one near 1450 nm (the first overtone of O–H bonds of water) and another around 1930–1940 nm (a combination band of O–H and C=O associated with water and carbohydrates)—are other prominent features of these spectra [23].

In contrast, adulterated samples show a broad decrease in reflectance (increase in absorption) in the 900–1800 nm range, which is particularly pronounced in the absorption bands for water and carbohydrate compounds (1450, 1930, and 1200 nm). This suggests a higher content of moisture and carbohydrate compounds in the common additives, such as corn powder (rich in starch) and citrus peel powders (containing cellulose and water), compared to genuine turmeric. At the higher end of the spectrum (above 2000 nm), adulterated samples exhibit slightly higher reflectance, which may be related to the unique characteristics of certain additives in this region.

The spectral differences observed between authentic and adulterated turmeric samples can be explained by the chemical composition of turmeric and the molecular structure of the adulterants. Authentic turmeric contains high levels of curcuminoids, particularly curcumin, which exhibit strong optical absorption in the visible region around 420–430 nm. When low-cost bulking agents such as corn flour or plant peel powders are added, the relative concentration of these pigments decreases, which can result in higher reflectance in the visible region. In the near-infrared region, the observed spectral variations are mainly associated with overtone and combination vibrations of chemical bonds such as O–H, C–H, and N–H. Adulterants rich in carbohydrates and starch (e.g., corn powder) exhibit characteristic spectral features related to O–H and C–H groups, particularly around 1200, 1450, and 1930 nm. Differences in chemical composition between authentic turmeric and adulterated samples therefore lead to noticeable variations in the reflectance spectra within these wavelength regions, which can be exploited as discriminative features for adulteration detection.

From a food safety perspective, the detection of such adulterants is highly important. While some bulking agents mainly reduce the nutritional quality of turmeric, the presence of synthetic dyes or chemical contaminants can pose serious health risks. Compounds such as Metanil Yellow and Sudan dyes have been associated with toxicological effects, including allergic reactions and potential carcinogenicity. Moreover, heavy-metal-based colorants such as lead chromate can cause neurological damage and kidney disorders. Therefore, the ability of spectroscopic techniques combined with machine learning to rapidly identify spectral signatures related to these compositional changes provides an important tool for protecting consumer health and improving food authenticity monitoring.

In this study, the performance of several machine learning algorithms for detecting authentic from counterfeit turmeric was investigated using spectral data in two ranges: 430–870 nm and 900–2170 nm. The results, presented in Table 1 and Table 2, include the models’ accuracy, precision, recall, and F1-score at three stages: training, validation, and testing. It should be noted that Table 1 and Table 2 present only the best results for each model, while the complete results for all model configurations are provided in the Supplementary Materials (Tables S2–S5). Furthermore, the performance of each model in each spectral range was analyzed separately, and finally, a comparison was made between the two ranges.

### 2.3. Results and Analysis of the 430–870 nm Spectral Range

In the decision tree model, two splitting criteria, Gini impurity (GDI) and deviance, were used at various depths. According to Table 1, the optimal DT configuration was achieved at a depth of 4 (deviance criterion), with a test accuracy of 0.944, indicating acceptable performance for this model. Higher depths did not improve the test accuracy and were therefore not selected, which could be a sign of potential overfitting. Thus, the deviance criterion with a moderate depth (MaxDepth = 4) was selected as the optimal combination among the decision tree models.

For the SVM model, three kernel types were evaluated: linear, radial basis function (RBF), and polynomial. The RBF kernel achieved the highest accuracy on the test data (100% accuracy, precision, recall, and F1-score, as shown in Table 1), demonstrating this method’s strong capability in class separation. However, its training accuracy was 0.8750, which is lower than the test accuracy. This discrepancy could be attributed to the model’s high generalization ability or the effects of random data partitioning. The polynomial kernel also showed performance comparable to the best models, with a test accuracy of 0.944 (consistent with Table 1).

The MLP model had a relatively lower performance compared to the other models, with a training accuracy of 0.8036 and a test accuracy of 0.889, but still showed good potential for classification, particularly if the network structure and parameters are further optimized.

This study investigated Quadratic LDA using 10–11 components (as reported in Table 1). The selected model reported training and test accuracies of 0.892–0.910 and 0.944, respectively. In addition to its high accuracy, this model demonstrated good stability across different stages and had lower complexity than the SVM model.

Based on Table 1, models such as SVM with the RBF kernel, Quadratic LDA with 10 or 11 components, and the decision tree with the deviance criterion at depth = 4 achieved a test accuracy of 0.944 or higher. Among these, the SVM with the RBF kernel showed a 100% test accuracy, but the relatively large difference between its training and test accuracy may suggest potential sensitivity to the data split. In contrast, the Quadratic LDA model, with its balanced performance in accuracy and stability, is proposed as the final recommendation for classifying authentic and adulterated turmeric within this spectral range.

The high accuracies achieved in this spectral range align with the results of similar research in food fraud detection. For instance, the study by Asadian et al. [24] (2024), which investigated the feasibility of Vis/NIR spectroscopy for detecting olive oil adulteration (with the addition of olive pomace oil) using LDA and SVM, achieved a training accuracy of 96.69% and a validation accuracy of 94.21% with the SVM model. In comparison, the achievement of 100% accuracy by SVM (RBF kernel) and 94.44% by LDA on the test data in the current study demonstrates the competitive, and even superior, performance of this approach in turmeric adulteration detection [24]. This difference could be due to the nature of the sample matrix (powder versus liquid) and the specific absorption characteristics of turmeric. Furthermore, regarding the detection of added colorants, the research by Di Anibal [25], which focused on identifying Sudan dyes in spices using UV–visible spectroscopy, highlights the importance of this spectral region for detecting colored compounds. Given that the current study also addresses the detection of synthetic colorants in turmeric, the high accuracies obtained confirm the capability of VIS/NIR spectroscopy in this domain and could offer a faster and more efficient method compared to traditional laboratory approaches [25].

### 2.4. Results and Analysis of the 900–2170 nm Spectral Range

To evaluate the performance of various machine learning models in differentiating authentic from counterfeit turmeric, DT, SVM, MLP, and LDA models were assessed using spectral data in the 900 to 2170 nm range. The results are presented in Table 2, and the complete results for all versions are included in Supplementary Tables S4 and S5.

The DT model achieved 100% accuracy across all configurations, including both GDI and deviance algorithms at various depths, for the training, validation, and test sets. This result indicates that the model completely learned the patterns within the data without overfitting, as evidenced by the consistent accuracy on the test data.

The SVM model also performed exceptionally well. The linear kernel achieved 100% accuracy on the training and validation data but experienced a drop in accuracy to 88.89% on the test set. In contrast, the RBF and Polynomial kernels both achieved 100% accuracy across all three datasets, demonstrating their high efficiency in class separation.

The MLP model showed strong performance as well, with accuracies of 98.21%, 100%, and 100% on the three datasets, confirming its capability in modeling nonlinear data.

For the LDA model, increasing the number of components significantly improved classification accuracy. At lower component numbers, accuracy was lower on the training and validation data. However, as the number of components increased, the test accuracy reached 100% at 2 and 3 components, and remained above 94% for most configurations. Therefore, linear LDA (as reported in Table 2) was selected as the final model for this range.

Based on the results in Table 2, it can be concluded that most of the models examined demonstrate high accuracy in classifying the samples. However, considering the stability of performance across the three datasets, the DT model, SVM with RBF and Polynomial kernels, and MLP are suggested as the superior options.

In a final summary, due to its perfect accuracy, high interpretability, and ease of implementation, the DT model is proposed as the best method for classifying authentic and counterfeit turmeric in the 900 to 2170 nm range.

The very high accuracies obtained in this spectral range (primarily in the NIR region) are in strong agreement with the findings of other studies that have utilized NIR spectroscopy for detecting food adulteration. For example, Agustami Sitorus et al.’s [26] research on detecting nutmeg adulteration with cinnamon using NIR spectroscopy also achieved a 100% classification accuracy with a PC-MLP model [26]. This similarity in final accuracies highlights the high discriminative power of NIR spectroscopy in identifying molecular patterns related to different chemical compounds in powdered spices. Additionally, in another study, Jiang et al. achieved 100% accuracy in identifying counterfeit edible oils using three-dimensional fluorescence spectroscopy combined with an MPCA-LDA approach. Although the spectroscopic tool is different, this study also emphasizes the exceptional ability of machine learning algorithms to achieve perfect accuracies in food adulteration detection [27]. The results of the current study within the NIR range confirm turmeric’s suitability as a product for implementing these advanced technologies and demonstrate that the excellent accuracies obtained in this field are not limited to a specific matrix or type of spectroscopy.

Table 3 summarizes previous research on adulteration detection in turmeric and related spices using spectroscopic techniques. According to this table, our proposed method achieves 100% test accuracy in the NIR range (900–2170 nm), which is higher than or comparable to previous studies. Notably, our study incorporates a hybrid feature selection method (SVM-LCA) that reduces data dimensionality and improves computational efficiency, a feature not employed in most prior works. Furthermore, our study evaluates a more diverse set of adulterants (4 bulking agents at 10–40% and 2 synthetic dyes at 0.5–3%) compared to most prior works, which typically focused on one or two adulterants. The perfect classification accuracy achieved in the NIR range demonstrates the high discriminative power of this spectral region for turmeric adulteration detection.

### 2.5. Selection of Effective Wavelengths

In this study, a combination of SVM (Support Vector Machine) and five common optimization and dimensionality reduction algorithms—League Championship Algorithm (LCA), Genetic Algorithm (GA), Particle Swarm Optimization (PSO), Ant Colony Optimization (ACO), and Imperialist Competitive Algorithm (ICA)—was used to extract effective features and reduce the dimensionality of turmeric’s spectral data. Each of these algorithms was evaluated based on two key spectral ranges: 430–870 nm and 900–2170 nm.

**Figure 3:**
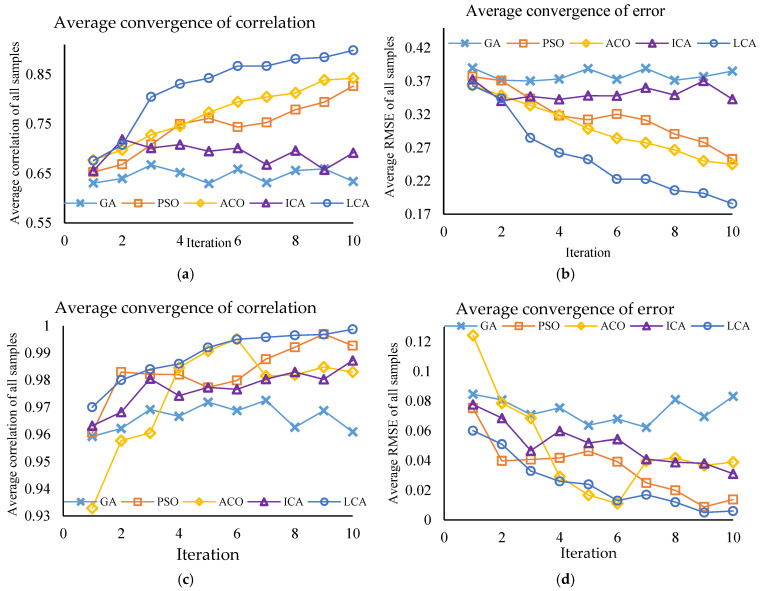
The performance of feature selection algorithms based on the average correlation and RMSE criteria. (**a**,**b**) represents the 430–870 nm range, while (**c**,**d**) represents the 900–2170 nm range.

The performance of the algorithms was assessed using key criteria, including mean correlation, mean RMSE (Root Mean Square Error), result stability across multiple iterations, and algorithm execution speed. Figure 3 illustrates the feature selection performance of these five algorithms based on mean correlation and mean RMSE over 10 iterations.

As shown in Figure 3, the LCA demonstrated the best performance in both spectral ranges by minimizing RMSE and maximizing the correlation coefficient. In the 430–870 nm range, the mean correlation coefficient for LCA was 0.82across various iterations, which was the highest value compared to other algorithms. Additionally, the algorithm’s RMSE decreased from 0.363 to 0.185, indicating an increase in accuracy and a reduction in error. In the 900–2170 nm range, LCA achieved a very high mean correlation coefficient (0.9894) and the lowest mean RMSE (0.02) among the algorithms.

Conversely, while the GA had a relatively good correlation coefficient, it showed higher RMSE values and greater instability in both ranges, which reduced confidence in its results. The PSO algorithm performed adequately and recorded a relatively low RMSE in the 900–2170 nm range, but its correlation coefficient and stability were lower than LCA’s in the 430–870 nm range. The ACO and ICA had moderate performance and could not approach the accuracy and stability of the LCA. In addition to accuracy and stability, the execution time of the algorithms was also considered. LCA demonstrated a suitable execution time, giving it an advantage over algorithms like ACO, which had longer runtimes.

Based on the combined criteria of accuracy, stability, and computational efficiency, the LCA was selected as the best dimensionality reduction method for turmeric’s spectral data. In total, 14 and 15 effective wavelengths were selected using this algorithm for the 430–870 nm and 900–2170 nm ranges, respectively. This selection allows for the use of key extracted wavelengths with the least error and highest correlation for subsequent modeling. Table 4 shows the wavelengths selected using the SVM-LCA method, which are included due to their significant impact. The wavelength selection analysis aimed to identify the most informative spectral regions contributing to sample classification. Reducing the number of wavelengths can decrease redundancy and noise in the data while improving computational efficiency. Furthermore, identifying a limited set of key wavelengths may facilitate the development of simplified and cost-effective spectroscopic devices for practical food authentication applications.

Consistent with the spectral interpretation in Figure 2, the selected wavelengths in the 900–2170 nm region (Table 4) correspond to specific overtone and combination vibrations of molecular bonds, providing a physicochemical basis for their discriminative power. The 905–1014 nm region is associated with third overtones of C–H stretching, primarily originating from curcuminoids and carbohydrates. The 1177–1268 nm region corresponds to C–H second overtones and C–H deformation combinations, which help distinguish differences in carbohydrate composition. The wavelengths at 1357 nm and 1412 nm are linked to N–H stretching and C–H combinations, which may arise from proteinaceous impurities in plant-based adulterants. The 1877–1911 nm region is characteristic of O–H stretching and bending combinations, strongly influenced by water content and starch-based adulterants. The 2087–2155 nm region corresponds to N–H and C=O combinations, which can differentiate turmeric from plant-based adulterants with different protein and carbohydrate profiles. In the 430–870 nm range, the selected wavelengths (e.g., 499, 543, 568, 585, 603, 617, 739, 784, 806, 827 nm) correspond to electronic absorption bands of curcuminoids and synthetic dyes, making this region particularly sensitive to color adulteration.

### 2.6. Classification Performance Based on Selected Wavelengths with the SVM-LCA

To improve classification efficiency and reduce computational complexity, feature selection was performed using the combined SVM-LCA in two spectral ranges: 430–870 nm and 900–2170 nm. After selecting the effective wavelengths, various classification models, including DT, SVM, MLP, and LDA, were retrained on the selected features. Their performance was evaluated in three stages: training, validation, and independent testing. Table 5 and Table 6 present only the optimal performance of each model, while the complete results of all model configurations are provided in the Supplementary Materials (Tables S5–S8).

### 2.7. Performance in the 430–870 nm Range

In the 430–870 nm range, even though the number of features was significantly reduced after applying SVM-LCA, most models maintained high and stable performance. As shown in Table 5, the DT model using a deviance criterion with a maximum depth of 10 achieved perfect classification performance on the test data (Accuracy, Precision, Recall and F1 = 1.00), with strong performance in training (Accuracy = 0.946) and validation (Accuracy = 0.947). Another DT configuration using the GDI criterion (MaxDepth = 4) also reached 100% test accuracy, demonstrating the robustness of simpler tree structures when combined with the selected features.

Among the SVM models, both the RBF and polynomial kernels achieved excellent performance. The polynomial kernel yielded high accuracy in training and validation (0.946 and 0.947, respectively) and reached 100% in all test metrics. The RBF kernel also achieved perfect results on the test set while showing slightly lower validation performance (Accuracy = 0.842). These results, also reflected in Supplementary Table S6, confirm the strong capability of SVM models in handling nonlinear boundaries when trained on the selected wavelengths.

For the MLP classifier, while validation performance was notably high (Accuracy = 0.947), its test accuracy decreased to 0.888, indicating some sensitivity to the dimensionality reduction. Among the LDA models (Table S7), configurations using 9–12 components showed the best results, achieving perfect accuracy in the test stage. For example, the 10-component model reached 1.00 for all test metrics, with balanced performance in training (Accuracy = 0.839) and validation (0.842). Models with fewer components showed a substantial performance decline, confirming the need for an adequate number of discriminant features in this spectral region.

Overall, in the 430–870 nm range, the best-performing models after SVM-LCA feature selection were the SVM with polynomial and RBF kernels, the Decision Tree (deviance, MaxDepth = 10), and Quadratic LDA with 9–10 components. Their 100% test accuracies highlight the successful extraction of the most informative wavelengths.

The accuracies obtained from classification with selected features in this spectral range not only demonstrate the efficacy of the SVM-LCA method in extracting critical information from spectral data but also align with the results of similar research in food fraud detection. For instance, the work by Du et al. on detecting raw milk adulterated with milk powder employed a similar approach using ATR-FTIR spectroscopy, machine learning, and feature selection techniques. They also achieved near-perfect classification performance with SVM and PLS-DA after feature selection, and at a low computational cost (Du, 2024) [28]. This consistency indicates that regardless of the specific type of spectroscopy, effective feature selection—like the SVM-LCA method in this study—can significantly enhance the performance of fraud detection models by reducing computational complexity while maintaining high accuracy. This is particularly important in the visible range, which contains information related to pigments and certain specific compounds. The 100% accuracies in our study highlight the high potential of this approach for the accurate detection of turmeric adulteration.

### 2.8. Performance in the 900–2170 nm Range

In the 900–2170 nm spectral range, the performance of all classifiers improved even further. As summarized in Table 6 and detailed in Supplementary Tables S8 and S9, the DT, SVM (linear kernel), and MLP models achieved perfect accuracy, precision, recall, and F1-score across all three stages (Train = Validation = Test = 1.00). These results demonstrate that the selected wavelengths in this range captured highly representative chemical information for distinguishing authentic and adulterated turmeric samples.

For the LDA models, even though training accuracy for models with fewer components (e.g., 3 components) was lower (Accuracy = 0.911), their validation and test accuracies remained high (0.947 and 1.00, respectively). The LDA configuration with 4 components delivered perfect performance in all metrics and datasets (Accuracy, Precision, Recall, and F1 = 1.00). This confirms that a small number of carefully selected wavelengths in the short-wave infrared range can fully preserve class separability.

Overall, the results from both spectral ranges demonstrate the high efficiency of the SVM-LCA in feature selection and in maintaining or even improving the classification performance of models compared to using the full spectral data. In both ranges, the use of wavelengths selected by the SVM-LCA algorithm either preserved or enhanced the performance of the classifiers.

Specifically, in the longer spectral range (900–2170 nm), the models showed almost ideal performance in all training, validation, and testing stages. On the other hand, in the shorter range (430–870 nm), although the accuracy of some models decreased slightly compared to using the full spectrum, a respectable accuracy was still observed.

Based on these results, we can conclude that the use of selected features not only reduces data dimensionality and increases processing speed but also maintains classification accuracy at a desirable level.

The exceptional 100% accuracies achieved in this spectral range with selected wavelengths align with previous achievements in the field of food fraud detection using spectroscopy and dimensionality reduction algorithms, confirming the power of the method. For example, the research by Zhao et al. [29] on the identification and quantification of adulteration in Chinese yam powder (a food powder similar to turmeric) used LIBS spectroscopy combined with chemometric methods, including variable optimization by PCA and Random Forest. They also achieved 100% classification accuracy with an RF-SVM model. This overlap in results shows the high capability of feature selection/extraction-based approaches in achieving impeccable detection in food powders [29]. The present study demonstrates that effective feature selection with SVM-LCA in the NIR range provides the necessary information for classifying turmeric with outstanding accuracy, which can help develop precise and efficient quality control systems. This is a significant step toward the industrial application of these methods.

### 2.9. Results

In this study, we performed accurate and non-destructive detection of genuine and counterfeit turmeric by utilizing full spectral data in two ranges—170–870 nm (UV/Vis) and 900–2170 nm (NIR)—and employing several machine learning algorithms including SVM, LDA, MLP, and DT. The spectral data in the 170–870 nm range were initially truncated at 430 nm due to noise. The results from applying machine learning methods to the full spectral data showed that the quadratic LDA model in the 430–870 nm range, with its high accuracy and stability, is the recommended method. Furthermore, in the 900–2170 nm range, the DT model achieved 100% accuracy across all stages, demonstrating the best performance and being selected as the final recommended model.

Optimized feature selection algorithms, including Imperialist Competitive Algorithm (ICA), Ant Colony Optimization (ACO), Particle Swarm Optimization (PSO), Genetic Algorithm (GA), and League Championship Algorithm (LCA), were used to reduce data dimensions and remove ineffective noise, which significantly improved the performance of the models. In the 430–870 nm range, the LCA showed suitable performance by reducing the Root Mean Square Error (RMSE) from 0.363 to 0.185 and increasing the average correlation coefficient to 0.82. In the 900–2170 nm range, this algorithm also achieved the best results with an average correlation coefficient of 0.9894 and an RMSE of 0.02. Based on this algorithm, 14 and 15 effective wavelengths were extracted from the VIS–NIR and NIR ranges, respectively.

To compare the results of modeling spectral data in the full range versus the selected effective wavelengths, machine learning methods were applied again. The classification models, including SVM, DT, MLP, and LDA, performed exceptionally well after being trained on the selected features. In the 430–870 nm range, the Decision Tree and SVM with a polynomial kernel both achieved 100% accuracy in the test set. Additionally, the LDA model with 9 to 14 components achieved 100% accuracy in the test set and approximately 95% in validation. In the 900–2170 nm range, all models, including SVM with linear and polynomial kernels, and MLP, successfully achieved 100% accuracy in all three stages: training, validation, and testing. Similarly, the LDA method (with 4 components) achieved 100% accuracy in the test and validation sets and 94.64% in the training set. These results indicate that the data in this spectral range contain richer information for sample discrimination.

### 2.10. Limitations and Future Perspectives

Although the proposed method achieved perfect or near-perfect classification for the adulterants tested, certain limitations should be acknowledged. First, no misclassifications were observed for models achieving 100% test accuracy (e.g., SVM with RBF kernel in the 900–2170 nm range). However, in the 430–870 nm range, the MLP model misclassified a small number of test samples at the lowest adulteration levels, indicating reduced sensitivity at very low adulterant concentrations. Second, the models were trained only on four specific bulking agents and two synthetic dyes. Performance on unknown adulterants (e.g., other plant powders, lead chromate, or Sudan dyes) has not been evaluated and may be significantly lower. Without retraining or model adaptation, the current classifiers cannot reliably detect unknown adulterants because they were trained as a closed-set classification problem. Third, all samples were prepared under laboratory conditions; therefore, generalization to blind commercial samples requires further validation. Specifically, we plan to collect at least 50 commercial turmeric samples from different retailers, analyze them with reference methods (HPLC for curcumin content and dye identification), and compare the spectroscopic predictions with ground-truth laboratory results. Such a validation step is essential before deploying the proposed method in routine quality control. Future work should explore anomaly detection frameworks (e.g., one-class SVM or autoencoders) to identify samples containing adulterants outside the training distribution (open-set scenarios). Additionally, expanding the adulterant library and testing on real-world commercial samples are necessary before industrial deployment.

## 3. Materials and Methods

### 3.1. Sample Preparation

In this study, a set of samples, including both authentic and adulterated turmeric, was prepared to simulate market fraud conditions and evaluate the capability of spectroscopy in detecting adulterated turmeric. Authentic turmeric samples were sourced from reputable suppliers. To ensure representativeness, the pure turmeric powders were collected from several well-known commercial brands and trusted local suppliers in Tehran, Iran, allowing us to capture the natural variability typically observed in consumer markets. Likewise, all adulterants—including corn powder, talc powder, orange peel powder, and pomegranate peel powder—were obtained from local commercial sources based on their documented and widespread use in turmeric adulteration. To create the adulterated samples, turmeric was combined with unauthorized additives, including corn powder, talc powder, orange peel powder, and pomegranate peel powder, each added at four different weight percentages: 10%, 20%, 30%, and 40%. Additionally, to simulate color adulteration, the synthetic dyes Tartrazine and Sunset Yellow were added to the samples at 0.5%, 1%, 2%, and 3% by weight. A total of 93 samples with various compositions were prepared to cover a wide range of common adulteration scenarios found in the market. The 93 samples comprised 21 pure turmeric samples (collected from 7 different commercial sources, 3 replicates each) and 72 adulterated samples. Each adulterant–concentration combination was prepared in 3 replicates. The detailed class distribution is provided in Supplementary Table S1.

Following uniform mixing, all samples were stored in sealed containers, protected from light and moisture, to preserve their physical properties throughout the experimental process.

A range of adulteration levels was intentionally prepared to simulate potential real-world adulteration scenarios. The selected concentration levels were chosen to represent plausible contamination levels reported in the literature and to evaluate the sensitivity of the proposed method across different degrees of adulteration.

### 3.2. Spectroscopy

For each sample, spectral data were recorded in two distinct ranges of the electromagnetic spectrum. The first range covered the 170 to 870 nm region, which includes the ultraviolet (UV) and visible (Vis) areas, while the second range encompassed the 900 to 2170 nm region, corresponding to the near-infrared (NIR) area. This division aligns with common standards in spectroscopy, where the UV region typically spans approximately 170 to 380 nm, the visible region from 380 to 780 nm (Ocean optic-usb2000, Dunedin, FL, USA), and the NIR region from 780 to 2500 nm. Therefore, the ranges selected for this study are fully in line with international standards. The specified spectrophotometer was configured to extract absorbance intensity data for each sample within the two aforementioned ranges, saving the data as Excel files for subsequent analysis. By simultaneously utilizing the UV/Vis and NIR regions, comprehensive and complete information on the optical behavior and chemical properties of the samples was obtained, forming the basis for subsequent classification and adulteration detection analyses.

### 3.3. Spectral Data Analysis

In this study, raw spectral data were collected in two ranges: 170–870 nm and 900–2170 nm, corresponding to the UV/Vis and NIR regions, respectively. The 170–870 nm range, specifically in the initial part of the spectrum from 170 nm to approximately 430 nm, exhibited considerable noise. This noise was attributed to a low signal-to-noise ratio and detector sensitivity limitations within this section. Consequently, the noisy data from this segment were removed, and only the data from 430 nm to 870 nm were used for subsequent analyses. After this initial correction, the spectral data were prepared for machine learning modeling.

For the modeling phase, four supervised learning algorithms were employed: Support Vector Machine (SVM), Linear Discriminant Analysis (LDA), Multilayer Perceptron (MLP) neural network, and Decision Tree (DT). The SVM algorithm was evaluated with three different kernels: linear, Radial Basis Function (RBF), and polynomial. This method was chosen for its ability to find optimal decision boundaries between classes in high-dimensional problems. The LDA algorithm was used as a classical discriminant method to create a linear combination of features that maximizes the separation between classes, and the impact of increasing components was also examined. The MLP neural network, with its multilayer structure consisting of three hidden layers with 10, 15, and 20 neurons, possessed the capability to identify complex non-linear patterns in the data and was utilized to enhance classification accuracy. Additionally, the Decision Tree algorithm, which operates based on sequential divisions of the feature space, was included to compare its performance with the other algorithms. In this method, Gini (gdi) and cross-entropy were used as criteria to evaluate the quality of splits at the nodes. Furthermore, the maximum number of splits (max splits) was investigated at three levels: 4, 10, and 20, to fine-tune the complexity of the tree structure.

All models were trained separately on the spectral data from both the UV/Vis and NIR ranges. To assess their generalization capability, each model was trained and validated 200 times using random data splits. The best split in terms of accuracy was selected. Classification Accuracy was used as the primary performance metric for comparing the algorithms.

### 3.4. Data Splitting and Model Evaluation

To build and evaluate machine learning models, the spectral data were divided into three main subsets: a training set, a validation set, and a test set. The data were split in a 60%, 20%, and 20% ratio, which is one of the most common splits used in machine learning problems, particularly with medium-sized datasets [30]. The training set was used to learn patterns, adjust coefficients, and optimize the model’s objective function. The validation set was used during model training to tune and optimize hyperparameters such as the kernel type in SVM, the number of components in LDA, the network structure in MLP, and the depth and branches of the tree in DT, playing a crucial role in preventing overfitting. The test set was exclusively used at the end of training to evaluate the model’s performance on unseen data and realistically assess its predictive accuracy.

To increase the accuracy of the results and reduce dependency on the initial random split, the training and evaluation process was performed in 200 repetitions for each model. In each repetition, the data were randomly split, and the modeling was re-executed, ultimately yielding the best classification accuracy. This iterative process not only ensures the stability of the models but also provides a realistic measure of their generalization ability. All modeling steps were implemented and executed in the MATLAB 2022 environment, and the performance of the classification models was evaluated using four standard metrics, including Accuracy, Precision, Recall, and F1-score.

### 3.5. Effective Wavelength Selection and Selected Feature Modeling

To enhance the efficiency of machine learning models and reduce computational load, feature selection and dimensionality reduction methods were employed. The initial spectral data, spanning the 170 to 870 nm range, contained sections with high noise, particularly at the beginning of the spectrum, which could negatively impact the quality of the modeling. Therefore, in the pre-modeling phase, data from high-noise regions up to a wavelength of 430 nm were removed to ensure more accurate and stable input data for the models. However, spectral data in the 900–2170 nm range were fully analyzed. Given the large number of spectral variables (wavelengths) and the potential for high correlation among them, selecting effective features for modeling was of particular importance. In this study, a combination of the SVM algorithm and five common optimization and dimensionality reduction algorithms—League Championship Algorithm (LCA), Genetic Algorithm (GA), Particle Swarm Optimization (PSO), Ant Colony Optimization (ACO), and Imperialist Competitive Algorithm (ICA)—was used to identify and select the optimal and influential wavelengths [31]. This method leverages SVM’s ability to differentiate data and, with the help of these five algorithms, intelligently identifies and selects the wavelengths that play the most significant role in separating genuine and counterfeit turmeric classes. The algorithms were evaluated for their performance based on mean correlation and RMSE over 10 iterations, and the best-performing one was chosen for subsequent modeling. This approach to dimensionality reduction was investigated for its ability to eliminate redundant and ineffective wavelengths, increase processing speed, and enhance the accuracy of classification models. The effective features (wavelengths) selected by the best algorithm were then used as input for various machine learning algorithms, including SVM, LDA, MLP, and Decision Tree. The performance of the models in the reduced-dimension state was compared with their performance in the initial state to evaluate the effectiveness of the feature selection. All data analysis, algorithm implementation, and classification modeling were performed using MATLAB version 2022, which is considered a powerful and standard environment for spectral data analysis and machine learning applications in engineering and agricultural sciences.

## 4. Conclusions

This research aimed to develop a rapid and non-destructive method for detecting adulteration in turmeric using UV–Vis and NIR spectroscopy combined with machine learning algorithms. The results demonstrated that this approach can serve as a powerful tool for ensuring the quality and authenticity of food products. The application of feature-selection optimization algorithms significantly reduced data dimensionality and improved the efficiency of the classification models.

The results further indicated that NIR spectroscopy provides richer information for sample discrimination, as all models developed using this spectral range achieved 100% classification accuracy. Compared with the performance obtained in the UV–Vis range, these results highlight the superior capability of NIR spectroscopy in identifying key chemical characteristics of turmeric samples. Therefore, the proposed method has strong potential to be adopted as an effective analytical tool in the food industry for detecting common forms of adulteration. Future studies may extend this approach to other spices and food products.

Although the measurements in this study were performed offline, the developed models can be readily integrated into online or inline spectroscopic systems for real-time adulteration detection.

Future work may include theoretical simulation of the near-infrared spectral features of curcumin using density functional theory (e.g., B3LYP) to further investigate the molecular origin of the observed spectral patterns and their influence on the spectra of pure and adulterated turmeric samples.

## Figures and Tables

**Table 1:** Optimal Performance of machine learning algorithms for identifying authentic and counterfeit turmeric samples in the 430–870 nm range.

Model	Configuration	Set	Accuracy	Precision	Recall	F1-Score
DT	Crt = gdi, MaxDepth = 10	Train	0.911	0.920	0.905	0.912
		Validation	0.842	0.835	0.840	0.837
		Test	0.944	0.945	0.940	0.942
	Crt = deviance, MaxDepth = 4	Train	0.875	0.880	0.870	0.875
		Validation	0.842	0.830	0.845	0.837
		Test	0.944	0.940	0.945	0.942
SVM	RBF kernel	Train	0.875	0.874	0.875	0.874
		Validation	0.947	0.944	0.954	0.946
		Test	1	1	1	1
MLP	-	Train	0.804	0.810	0.795	0.802
		Validation	0.895	0.887	0.895	0.89
		Test	0.889	0.885	0.890	0.887
Quadratic LDA	Components = 10	Train	0.892	0.894	0.895	0.892
		Validation	1	0.995	1	0.997
		Test	0.944	0.940	0.945	0.942
	Components = 11	Train	0.910	0.905	0.913	0.907
		Validation	0.947	0.942	0.952	0.945
		Test	0.944	0.945	0.945	0.942

**Table 2:** Optimal Performance of machine learning algorithms for identifying genuine and adulterated turmeric samples in the 900–2170 nm range.

Model	Configuration	Set	Accuracy	Precision	Recall	F1-Score
DT	Crt = gdi, MaxDepth = 4	Train	1	1	1	1
		Validation	1	1	1	1
		Test	1	1	1	1
	Crt = deviance, MaxDepth = 4	Train	1	1	1	1
		Validation	1	1	1	1
		Test	1	1	1	1
SVM	RBF kernel	Train	0.982	0.979	0.984	0.981
		Validation	1	1	1	1
		Test	1	1	1	1
	Polynomial kernel	Train	1	1	1	1
		Validation	1	1	1	1
		Test	1	1	1	1
MLP	-	Train	0.982	0.985	0.98	0.982
		Validation	1	1	1	1
		Test	1	1	1	1
linear LDA	Components = 2	Train	0.893	0.895	0.890	0.892
		Validation	0.895	0.890	0.895	0.892
		Test	1	1	1	1
	Components = 3	Train	0.946	0.945	0.948	0.946
		Validation	1	1	1	1
		Test	1	1	1	1
	Components = 4	Train	0.946	0.945	0.948	0.946
		Validation	0.895	0.890	0.895	0.892
		Test	0.944	0.942	0.945	0.944

**Table 3:** Comparison of the proposed method with previous studies for adulteration detection in turmeric and related spices.

Study	Spectroscopy	Chemometric Method	Adulterants Tested	Test Accuracy
[9]	NIR	PLSR, PCR	Metanil Yellow (1–25%)	PLSR: R^2^_p_ = 0.974, RMSEP = 0.725%
[15]	FT-NIR	SIMCA, PLSR	Sudan Red, Metanil Yellow (1–25%)	SIMCA: 93.4% validation; PLSR: R^2^ = 0.90–0.91
[7]	Vis-NIR	Linear Regression, SVM	Spent turmeric (0–100%)	Linear Regression: error < 15%
[16]	FT-NIR	RF, GB, SVM	Corn starch, soybean flour, wheat flour (10–50%)	RF and GB: 100%
[13]	Raman FT-IR	1D-CNN, RF, sPLS-DA, PC-LDA	Lead chromate, Metanil Yellow, Orange II, Sudan III, Starch (1–100%)	1D-CNN: 97%; RF: 93%
This study	UV/Vis + NIR	SVM, DT, MLP, LDA	4 bulking agents + 2 synthetic dyes (10–40%, 0.5–3%)	100% (NIR range)

**Table 4:** Effective wavelengths selected by the SVM-LCA method.

Range (nm)	Selected EWs (nm)
430–870	499.195, 784.297, 617.938, 478.212, 490.874, 543.535, 739.379, 522.649, 568.333, 827.007, 806.688, 585.194, 603.286, 530.884
900–2170	1055.73, 1877.77, 1357.27, 1911.56, 991.872, 2119.19, 1412.28, 2087.79, 2155.43, 1014.41, 1177.14, 1184.65, 1268.47, 905.445, 2115.53

**Table 5:** Optimal Performance of machine learning algorithms for identifying authentic and counterfeit turmeric samples in the 430–870 nm range using effective wavelengths.

Model	Configuration	Set	Accuracy	Precision	Recall	F1-Score
DT	Crt = gdi, MaxDepth = 4	Train	0.929	0.93	0.928	0.929
		Validation	0.895	0.89	0.895	0.892
		Test	1	1	1	1
	Crt = deviance, MaxDepth = 10	Train	0.946	0.948	0.945	0.946
		Validation	0.947	0.945	0.95	0.947
		Test	1	1	1	1
SVM	RBF kernel	Train	0.911	0.915	0.91	0.912
		Validation	0.842	0.835	0.845	0.840
		Test	1	1	1	1
	Polynomial kernel	Train	0.946	0.948	0.945	0.946
		Validation	0.947	0.945	0.950	0.947
		Test	1	1	1	1
MLP	-	Train	0.839	0.840	0.838	0.839
		Validation	0.947	0.945	0.950	0.947
		Test	0.888	0.883	0.883	0.883
Quadratic LDA	Components = 9	Train	0.821	0.82	0.825	0.822
		Validation	0.789	0.785	0.79	0.787
		Test	1	1	1	1
	Components = 10	Train	0.839	0.84	0.835	0.837
		Validation	0.842	0.84	0.845	0.842
		Test	1	1	1	1

**Table 6:** Optimal Performance of machine learning algorithms for identifying genuine and adulterated turmeric samples in the 900–2170 nm range using effective wavelengths.

Model	Configuration	Set	Accuracy	Precision	Recall	F1-Score
DT	Crt = gdi, MaxDepth = 4	Train	1	1	1	1
		Validation	1	1	1	1
		Test	1	1	1	1
	Crt = deviance, MaxDepth = 4	Train	1	1	1	1
		Validation	1	1	1	1
		Test	1	1	1	1
SVM	Linear kernel	Train	1	1	1	1
		Validation	1	1	1	1
		Test	1	1	1	1
MLP	-	Train	1	1	1	1
		Validation	1	1	1	1
		Test	1	1	1	1
linear LDA	Components = 3	Train	0.911	0.912	0.91	0.911
		Validation	0.947	0.945	0.95	0.947
		Test	1	1	1	1
	Components = 4	Train	0.946	0.945	0.947	0.946
		Validation	1	1	1	1
		Test	1	1	1	1

## Data Availability

The original contributions presented in this study are included in the article/Supplementary Materials. Further inquiries can be directed to the corresponding authors.

## Supplementary Materials

The following supporting information can be downloaded at: https://www.mdpi.com/article/10.3390/molecules31101774/s1, Table S1. Distribution of pure and adulterated turmeric samples across classes; Table S2. Complete performance of machine learning algorithms for identifying authentic and adulterated turmeric samples in the 430–870 nm range before effective wavelength selection; Table S3. Complete performance of the Quadratic LDA algorithm for identifying authentic and adulterated turmeric samples in the 430–870 nm range before effective wavelength selection; Table S4. Complete performance of machine learning algorithms for identifying authentic and adulterated turmeric samples in the 900–2170 nm range before effective wavelength selection; Table S5. Complete performance of the Linear LDA algorithm for identifying authentic and adulterated turmeric samples in the 900–2170 nm range before effective wavelength selection; Table S6. Complete performance of machine learning algorithms for identifying authentic and adulterated turmeric samples in the 430–870 nm range after effective wavelength selection; Table S7. Complete performance of the Quadratic LDA algorithm for identifying authentic and adulterated turmeric samples in the 430–870 nm range after effective wavelength selection; Table S8. Complete performance of machine learning algorithms for identifying authentic and adulterated turmeric samples in the 900–2170 nm range after effective wavelength selection; Table S9. Complete performance of the Linear LDA algorithm for identifying authentic and adulterated turmeric samples in the 900–2170 nm range after effective wavelength selection.

## References

[1] Kocaadam B., Şanlier N. (2017). Curcumin, an active component of turmeric (*Curcuma longa*), and its effects on health. Crit. Rev. Food Sci. Nutr..

[2] Chattopadhyay I., Biswas K., Bandyopadhyay U., Banerjee R.K. (2004). Turmeric and curcumin: Biological actions and medicinal applications. Curr. Sci..

[3] Tamiji Z., Habibi Z., Pourjabbar Z., Khoshayand M.R., Sadeghi N., Hajimahmoodi M. (2022). Detection and quantification of adulteration in turmeric by spectroscopy coupled with chemometrics. J. Consum. Prot. Food Saf..

[4] Yang H.J., Lim S., Lee D.H., Yun C.I., Kim Y.J. (2024). Validation and measurement uncertainty of analytical methods for various azo dye adulterants in *Curcuma longa* L.. J. Food Sci..

[5] Lanjewar M.G., Morajkar P.P., Parab J.S. (2024). Portable system to detect starch adulteration in turmeric using NIR spectroscopy. Food Control.

[6] Kar S., Tudu B., Bandyopadhyay R. (2024). Statistical machine learning techniques applied to NIR spectral data for rapid detection of sudan dye-I in turmeric powders with optimized pre-processing and wavelength selection. J. Food Sci. Technol..

[7] Behera A.R., Suresh H., Kumar A., Selvaraja S.K., Pratap R. Detection of spent turmeric adulteration in powdered *Curcuma longa* using Vis-NIR spectroscopy and machine learning. Proceedings of the 2020 5th IEEE International Conference on Emerging Electronics (ICEE).

[8] De Angelis E., Al-Ayoubi O., Pilolli R., Monaci L., Bejjani A. (2024). Time-of-flight secondary ion mass spectrometry coupled with unsupervised methods for advanced saffron authenticity screening. Foods.

[9] Kar S., Tudu B., Bag A.K., Bandyopadhyay R. (2018). Application of near-infrared spectroscopy for the detection of metanil yellow in turmeric powder. Food Anal. Methods.

[10] Velázquez R., Rodríguez A., Hernández A., Casquete R., Benito M.J., Martín A. (2023). Spice and herb frauds: Types, incidence, and detection: The state of the art. Foods.

[11] Schumer N.G., Ahmed M.W., Rausch K., Singh V., Kamruzzaman M. (2025). Chemometric-based approach for economically motivated fraud detection in organic spices via NIR spectroscopy. J. Food Compos. Anal..

[12] Sadeghi A., Khani S., Sabourian R., Hajimahmoodi M., Ghasemi J.B. (2025). Integrating CNNs and chemometrics for analyzing NIR spectra and RGB images in turmeric adulterant detection. J. Food Compos. Anal..

[13] Teklemariam T.A. (2024). Raman and Mid-Infrared Spectroscopy Coupled With Machine-Deep Learning for Adulterant Detection in Ground Turmeric. Appl. Spectrosc. Pract..

[14] Kar S., Tudu B., Bandyopadhyay R., Bag A.K. Discrimination of turmeric brands by means of near infrared (NIR) spectroscopy combined with chemometrics. Proceedings of the 2016 International Conference on Intelligent Control Power and Instrumentation (ICICPI).

[15] Khodabakhshian R., Bayati M.R., Emadi B. (2022). Adulteration detection of Sudan Red and metanil yellow in turmeric powder by NIR spectroscopy and chemometrics: The role of preprocessing methods in analysis. Vib. Spectrosc..

[16] Yu D.-X., Guo S., Zhang X., Yan H., Zhang Z.-Y., Chen X., Chen J.-Y., Jin S.-J., Yang J., Duan J.-A. (2022). Rapid detection of adulteration in powder of ginger (Zingiber officinale Roscoe) by FT-NIR spectroscopy combined with chemometrics. Food Chem. X.

[17] Cottenet G., Andrey D., Dubascoux S. (2024). Evaluation of ED-XRF for the detection of inorganic adulterants in turmeric, paprika and oregano. Food Addit. Contam. Part A.

[18] Kar S., Tudu B., Jana A., Bandyopadhyay R. (2019). FT-NIR spectroscopy coupled with multivariate analysis for detection of starch adulteration in turmeric powder. Food Addit. Contam. Part A.

[19] Petrakis E.A., Polissiou M.G. (2017). Assessing saffron (*Crocus sativus* L.) adulteration with plant-derived adulterants by diffuse reflectance infrared Fourier transform spectroscopy coupled with chemometrics. Talanta.

[20] Jariah A., Hadisaputra S., Purwoko A.A. (2023). Characterization of M-curcumin complexes (M = Cu, Co, Ag) in turmeric rhizome as sensitizer candidates in dye-sensitized solar cell (DSSC). Acta Chim. Asiana.

[21] Rajkumar D., Künnemeyer R., Kaur H., Longdell J., McGlone A. (2022). Interactions of linearly polarized and unpolarized light on kiwifruit using aquaphotomics. Molecules.

[22] Curra A., Gasbarrone R., Gattabria D., Bonifazi G., Serranti S., Greco D., Missori P., Fattapposta F., Picciano A., Maffucci A. (2024). In Vivo Insights: Near-Infrared Photon Sampling of Reflectance Spectra from Cranial and Extracranial Sites in Healthy Individuals and Patients with Essential Tremor. Photonics.

[23] Sundaram J., Mani S., Kandala C.V., Holser R.A. (2015). Application of NIR reflectance spectroscopy on rapid determination of moisture content of wood pellets. Am. J. Anal. Chem..

[24] Asadian S., Banakar A., Jamshidi B. (2024). Feasibility of Visible/Near Infrared Spectroscopy in order to detect pomace olive oil fraud with LDA and SVM detection methods. Innov. Food Technol..

[25] Di Anibal C.V. (2011). Determination of Banned Sudan Dyes in Culinary Spices Through Spectroscopic Techniques and Multivariate Analysis. Ph.D. Thesis.

[26] Sitorus A., Pambudi S., Boodnon W., Lapcharoensuk R. (2024). Near-infrared spectroscopy with machine learning for classifying and quantifying nutmeg adulteration. Anal. Lett..

[27] Jiang H., Cui Y., Jia Y. (2024). Edible Oil Identification Technology Based on Three-Dimensional Fluorescence Spectroscopy and MPCA-LDA. IEEE Access.

[28] Du L. (2024). New insights into raw milk adulterated with milk powder identification: ATR-FTIR spectroscopic fingerprints combined with machine learning and feature selection approaches. J. Food Compos. Anal..

[29] Zhao Z., Wang Q., Xu X., Chen F., Teng G., Wei K., Chen G., Cai Y., Guo L. (2022). Accurate identification and quantification of Chinese yam powder adulteration using laser-induced breakdown spectroscopy. Foods.

[30] Muraina I. Ideal dataset splitting ratios in machine learning algorithms: General concerns for data scientists and data analysts. Proceedings of the 7th International Mardin Artuklu Scientific Research Conference.

[31] Masoudi-Sobhanzadeh Y., Motieghader H., Masoudi-Nejad A. (2019). FeatureSelect: A software for feature selection based on machine learning approaches. BMC Bioinform..

