# Supplementary material for: Machine Learning and the Use of Spectroscopy for Adulteration Detection in Turmeric Powder
Source: Molecules. 2026 May 21;31(10):1774. doi: 10.3390/molecules31101774 (PMC13209246; doi:10.3390/molecules31101774)
Supplement: Supplementary file 1 [file molecules-31-01774-s001.zip › molecules-4184586-supplementary.pdf]

Table S1. Distribution of pure and adulterated turmeric samples across classes.

Sample Category	Adulterant	Concentration (% w/w)	Replicates	Total
Pure	-	-	21	21
Adulterated (bulking agents)	Corn powder	10, 20, 30, 40	3 × 4 = 12	12
Adulterated (bulking agents)	Talc powder	10, 20, 30, 40	3 × 4 = 12	12
Adulterated (bulking agents)	Orange peel powder	10, 20, 30, 40	3 × 4 = 12	12
Adulterated (bulking agents)	Pomegranate peel powder	10, 20, 30, 40	3 × 4 = 12	12
Adulterated (dyes)	Tartrazine	0.5, 1, 2, 3	3 × 4 = 12	12
Adulterated (dyes)	Sunset Yellow	0.5, 1, 2, 3	3 × 4 = 12	12
Total				93

Table S2. Complete performance of machine learning algorithms for identifying authentic and counterfeit turmeric samples in the 430-870 nm range before effective wavelengths selection

Model	Configuration	Set	Accuracy	Precision	Recall	F1-score
DT	Crt = gdi, MaxDepth = 4	Train	0.911	0.915	0.905	0.910
		Validation	0.842	0.835	0.840	0.837
		Test	0.889	0.890	0.885	0.887
	Crt = gdi, MaxDepth = 10	Train	0.911	0.920	0.905	0.912
		Validation	0.842	0.835	0.840	0.837
		Test	0.944	0.945	0.940	0.942
	Crt = gdi, MaxDepth = 20	Train	0.911	0.915	0.905	0.910
		Validation	0.895	0.890	0.895	0.892
		Test	0.944	0.943	0.945	0.944
	Crt = deviance, MaxDepth = 4	Train	0.875	0.880	0.870	0.875
		Validation	0.842	0.830	0.845	0.837
		Test	0.944	0.940	0.945	0.942
	Crt = deviance, MaxDepth = 10	Train	0.929	0.930	0.925	0.927
		Validation	0.842	0.835	0.840	0.837
		Test	0.944	0.945	0.940	0.942
	Crt = deviance, MaxDepth = 20	Train	0.964	0.965	0.960	0.962
		Validation	0.895	0.890	0.895	0.892
		Test	0.889	0.885	0.890	0.887
SVM	Linear kernel	Train	0.946	0.954	0.942	0.945
		Validation	1	1	1	1
		Test	0.888	0.883	0.883	0.883
	RBF kernel	Train	0.875	0.874	0.875	0.874
		Validation	0.947	0.944	0.954	0.946
		Test	1	1	1	1
	Polynomial kernel	Train	0.928	0.928	0.930	0.928
		Validation	1	1	1	1
		Test	0.944	0.0950	0.946	0.946
MLP	-	Train	0.804	0.810	0.795	0.802
		Validation	0.895	0.887	0.895	0.89
		Test	0.889	0.885	0.890	0.887

Table S3. Complete performance of Quadratic LDA algorithm for identifying authentic and counterfeit turmeric samples in the 430-870 nm range before effective wavelengths selection

Model	Configuration	Set	Accuracy	Precision	Recall	F1-score
Quadratic LDA	Components = 1	Train	0.571	0.562	0.571	0.565
		Validation	0.578	0.575	0.585	0.577
		Test	0.555	0.553	0.555	0.553
	Components = 2	Train	0.589	0.585	0.59	0.587
		Validation	0.684	0.675	0.684	0.677
		Test	0.555	0.564	0.553	0.555
	Components = 3	Train	0.678	0.687	0.675	0.677
		Validation	0.894	0.896	0.895	0.892
		Test	0.777	0.775	0.780	0.777
	Components = 4	Train	0.732	0.735	0.734	0.732
		Validation	0.947	0.940	0.952	0.945
		Test	0.888	0.885	0.890	0.887
	Components = 5	Train	0.714	0.713	0.715	0.712
		Validation	0.947	0.945	0.950	0.945
		Test	0.888	0.885	0.895	0.887
	Components = 6	Train	0.803	0.804	0.805	0.802
		Validation	0.947	0.940	0.951	0.945
		Test	0.944	0.941	0.945	0.942
	Components = 7	Train	0.767	0.765	0.770	0.767
		Validation	0.894	0.890	0.895	0.892
		Test	0.944	0.940	0.945	0.942
	Components = 8	Train	0.803	0.801	0.805	0.802
		Validation	0.894	0.890	0.895	0.892
		Test	0.944	0.941	0.945	0.942
	Components = 9	Train	0.839	0.835	0.840	0.837
		Validation	0.947	0.944	0.950	0.945
		Test	0.944	0.943	0.945	0.942
	Components = 10	Train	0.892	0.894	0.895	0.892
		Validation	1	0.995	1	0.997
		Test	0.944	0.940	0.945	0.942
	Components = 11	Train	0.910	0.905	0.913	0.907
		Validation	0.947	0.942	0.952	0.945
		Test	0.944	0.945	0.945	0.942
	Components = 12	Train	0.946	0.947	0.945	0.942
		Validation	0.947	0.944	0.950	0.945
		Test	0.944	0.943	0.945	0.942
	Components = 13	Train	0.946	0.947	0.945	0.942
		Validation	0.947	0.943	0.950	0.945
		Test	0.944	0.944	0.945	0.942
	Components = 14	Train	0.946	0.945	0.945	0.942
		Validation	0.947	0.946	0.957	0.945
		Test	0.944	0.942	0.945	0.942
	Components = 15	Train	0.946	0.945	0.945	0.942
		Validation	0.947	0.947	0.954	0.945
		Test	0.944	0.943	0.945	0.942

Table S4. Complete performance of machine learning algorithms for identifying genuine and adulterated turmeric samples in the 900–2170 nm range before effective wavelengths selection

Model	Configuration	Set	Accuracy	Precision	Recall	F1-score
**DT**	Crt = gdi, MaxDepth = 4	Train	1	1	1	1
		Validation	1	1	1	1
		Test	1	1	1	1
	Crt = gdi, MaxDepth = 10	Train	1	1	1	1
		Validation	1	1	1	1
		Test	1	1	1	1
	Crt = gdi, MaxDepth = 20	Train	1	1	1	1
		Validation	1	1	1	1
		Test	1	1	1	1
	Crt = deviance, MaxDepth = 4	Train	1	1	1	1
		Validation	1	1	1	1
		Test	1	1	1	1
	Crt = deviance, MaxDepth = 10	Train	1	1	1	1
		Validation	1	1	1	1
		Test	1	1	1	1
	Crt = deviance, MaxDepth = 20	Train	1	1	1	1
		Validation	1	1	1	1
		Test	1	1	1	1
**SVM**	Linear kernel	Train	1	1	1	1
		Validation	1	1	1	1
		Test	0.889	0.887	0.887	0.887
	RBF kernel	Train	0.982	0.979	0.984	0.981
		Validation	1	1	1	1
		Test	1	1	1	1
	Polynomial kernel	Train	1	1	1	1
		Validation	1	1	1	1
		Test	1	1	1	1
MLP	-	Train	0.982	0.985	0.98	0.982
		Validation	1	1	1	1
		Test	1	1	1	1

Table S5. Complete performance of linear LDA algorithm for identifying genuine and adulterated turmeric samples in the 900–2170 nm range before effective wavelengths selection

Model	Configuration	Set	Accuracy	Precision	Recall	F1-score
linear LDA	Components = 1	Train	0.821	0.825	0.820	0.822
		Validation	0.842	0.840	0.845	0.842
		Test	0.944	0.942	0.945	0.944
	Components = 2	Train	0.893	0.895	0.890	0.892
		Validation	0.895	0.890	0.895	0.892
		Test	1	1	1	1
	Components = 3	Train	0.946	0.945	0.948	0.946
		Validation	1	1	1	1
		Test	1	1	1	1
	Components = 4	Train	0.946	0.945	0.948	0.946
		Validation	0.895	0.890	0.895	0.892
		Test	0.944	0.942	0.945	0.944
	Components = 5	Train	0.893	0.895	0.890	0.892
		Validation	0.895	0.890	0.895	0.892
		Test	0.944	0.942	0.945	0.944
	Components = 6	Train	0.929	0.930	0.928	0.929
		Validation	0.895	0.890	0.895	0.892
		Test	0.944	0.942	0.945	0.944
	Components = 7	Train	0.929	0.930	0.928	0.929
		Validation	0.895	0.890	0.895	0.892
		Test	0.944	0.942	0.945	0.944
	Components = 8	Train	0.929	0.934	0.928	0.929
		Validation	0.895	0.893	0.895	0.892
		Test	0.944	0.942	0.945	0.944
	Components = 9	Train	0.929	0.933	0.928	0.929
		Validation	0.947	0.945	0.945	0.945
		Test	0.944	0.942	0.945	0.944
	Components = 10	Train	0.911	0.910	0.915	0.912
		Validation	0.895	0.890	0.895	0.892
		Test	0.944	0.942	0.945	0.944
	Components = 11	Train	0.946	0.945	0.948	0.946
		Validation	0.842	0.835	0.845	0.840
		Test	0.944	0.942	0.945	0.944
	Components = 12	Train	0.929	0.930	0.928	0.929
		Validation	0.842	0.835	0.845	0.840
		Test	0.944	0.942	0.945	0.944
	Components = 13	Train	0.964	0.965	0.960	0.962
		Validation	0.895	0.890	0.895	0.892
		Test	0.944	0.942	0.945	0.944
	Components = 14	Train	0.964	0.965	0.960	0.962
		Validation	0.842	0.835	0.845	0.84
		Test	0.944	0.942	0.945	0.944
	Components = 15	Train	0.982	0.985	0.981	0.982
		Validation	0.842	0.835	0.845	0.841
		Test	0.944	0.942	0.945	0.944

Table S6. Complete performance of machine learning algorithms for identifying authentic and counterfeit turmeric samples in the 430-870 nm range after effective wavelengths selection

Model	Configuration	Set	Accuracy	Precision	Recall	F1-score
DT	Crt = gdi, MaxDepth = 4	Train	0.929	0.93	0.928	0.929
		Validation	0.895	0.89	0.895	0.892
		Test	1	1	1	1
	Crt = gdi, MaxDepth = 10	Train	0.929	0.93	0.928	0.929
		Validation	0.947	0.945	0.95	0.947
		Test	0.944	0.942	0.945	0.944
	Crt = gdi, MaxDepth = 20	Train	0.929	0.93	0.928	0.929
		Validation	0.947	0.945	0.95	0.947
		Test	0.944	0.942	0.945	0.944
	Crt = deviance, MaxDepth = 4	Train	0.911	0.915	0.91	0.912
		Validation	1	1	1	1
		Test	0.944	0.942	0.945	0.944
	Crt = deviance, MaxDepth = 10	Train	0.946	0.948	0.945	0.946
		Validation	0.947	0.945	0.95	0.947
		Test	1	1	1	1
	Crt = deviance, MaxDepth = 20	Train	0.946	0.948	0.945	0.946
		Validation	0.895	0.89	0.895	0.892
		Test	1	1	1	1
SVM	Linear kernel	Train	0.804	0.81	0.8	0.805
		Validation	1	1	1	1
		Test	0.889	0.883	0.883	0.883
	RBF kernel	Train	0.911	0.915	0.91	0.912
		Validation	0.842	0.835	0.845	0.840
		Test	1	1	1	1
	Polynomial kernel	Train	0.946	0.948	0.945	0.946
		Validation	0.947	0.945	0.950	0.947
		Test	1	1	1	1
MLP	-	Train	0.839	0.840	0.838	0.839
		Validation	0.947	0.945	0.950	0.947
		Test	0.888	0.883	0.883	0.883

Table S7. Complete performance of Quadratic LDA algorithm for identifying authentic and counterfeit turmeric samples in the 430-870 nm range after effective wavelengths selection

Model	Configuration	Set	Accuracy	Precision	Recall	F1-score
Quadratic LDA	Components = 1	Train	0.554	0.56	0.555	0.557
		Validation	0.474	0.47	0.475	0.472
		Test	0.389	0.38	0.39	0.385
	Components = 2	Train	0.643	0.645	0.64	0.642
		Validation	0.421	0.42	0.425	0.422
		Test	0.444	0.44	0.445	0.442
	Components = 3	Train	0.768	0.77	0.765	0.767
		Validation	0.789	0.785	0.79	0.787
		Test	0.833	0.83	0.835	0.832
	Components = 4	Train	0.714	0.71	0.715	0.712
		Validation	0.684	0.68	0.685	0.682
		Test	0.889	0.885	0.89	0.887
	Components = 5	Train	0.732	0.735	0.73	0.732
		Validation	0.789	0.785	0.79	0.787
		Test	0.833	0.83	0.835	0.832
	Components = 6	Train	0.75	0.748	0.752	0.75
		Validation	0.737	0.735	0.74	0.737
		Test	0.889	0.885	0.89	0.887
	Components = 7	Train	0.821	0.82	0.825	0.822
		Validation	0.737	0.735	0.74	0.737
		Test	0.944	0.942	0.945	0.944
	Components = 8	Train	0.804	0.8	0.805	0.802
		Validation	0.737	0.735	0.74	0.737
		Test	0.889	0.885	0.89	0.887
	Components = 9	Train	0.821	0.82	0.825	0.822
		Validation	0.789	0.785	0.79	0.787
		Test	1	1	1	1
	Components = 10	Train	0.839	0.84	0.835	0.837
		Validation	0.842	0.84	0.845	0.842
		Test	1	1	1	1
	Components = 11	Train	0.893	0.895	0.89	0.892
		Validation	0.842	0.84	0.845	0.842
		Test	1	1	1	1
	Components = 12	Train	0.946	0.948	0.945	0.946
		Validation	0.895	0.89	0.895	0.892
		Test	1	1	1	1
	Components = 13	Train	0.964	0.965	0.96	0.962
		Validation	0.895	0.89	0.895	0.892
		Test	0.944	0.942	0.945	0.944
	Components = 14	Train	0.964	0.965	0.96	0.962
		Validation	0.947	0.945	0.95	0.947
		Test	0.944	0.942	0.945	0.944

Table S8. Complete performance of machine learning algorithms for identifying genuine and adulterated turmeric samples in the 900–2170 nm range after effective wavelengths selection

Model	Configuration	Set	Accuracy	Precision	Recall	F1-score
**DT**	Crt = gdi, MaxDepth = 4	Train	1	1	1	1
		Validation	1	1	1	1
		Test	1	1	1	1
	Crt = gdi, MaxDepth = 10	Train	1	1	1	1
		Validation	1	1	1	1
		Test	1	1	1	1
	Crt = gdi, MaxDepth = 20	Train	1	1	1	1
		Validation	1	1	1	1
		Test	1	1	1	1
	Crt = deviance, MaxDepth = 4	Train	1	1	1	1
		Validation	1	1	1	1
		Test	1	1	1	1
	Crt = deviance, MaxDepth = 10	Train	1	1	1	1
		Validation	1	1	1	1
		Test	1	1	1	1
	Crt = deviance, MaxDepth = 20	Train	1	1	1	1
		Validation	1	1	1	1
		Test	1	1	1	1
**SVM**	Linear kernel	Train	1	1	1	1
		Validation	1	1	1	1
		Test	1	1	1	1
	RBF kernel	Train	0.946	0.945	0.947	0.946
		Validation	1	1	1	1
		Test	1	1	1	1
	Polynomial kernel	Train	1	1	1	1
		Validation	1	1	1	1
		Test	1	1	1	1
**MLP**	-	Train	1	1	1	1
		Validation	1	1	1	1
		Test	1	1	1	1

Table S9. Complete performance of linear LDA algorithm for identifying genuine and adulterated turmeric samples in the 900–2170 nm range after effective wavelengths selection

Model	Configuration	Set	Accuracy	Precision	Recall	F1-score
linear LDA	Components = 1	Train	0.946	0.947	0.944	0.946
		Validation	1	1	1	1
		Test	0.944	0.942	0.945	0.944
	Components = 2	Train	0.911	0.91	0.912	0.911
		Validation	1	1	1	1
		Test	0.944	0.945	0.942	0.944
	Components = 3	Train	0.911	0.912	0.91	0.911
		Validation	0.947	0.945	0.95	0.947
		Test	1	1	1	1
	Components = 4	Train	0.946	0.945	0.947	0.946
		Validation	1	1	1	1
		Test	1	1	1	1
	Components = 5	Train	0.857	0.855	0.86	0.857
		Validation	0.895	0.892	0.897	0.895
		Test	1	1	1	1
	Components = 6	Train	0.893	0.892	0.895	0.893
		Validation	1	1	1	1
		Test	0.944	0.947	0.942	0.944
	Components = 7	Train	0.893	0.895	0.892	0.893
		Validation	0.895	0.897	0.892	0.895
		Test	1	1	1	1
	Components = 8	Train	0.875	0.877	0.872	0.875
		Validation	0.947	0.945	0.95	0.947
		Test	1	1	1	1
	Components = 9	Train	0.875	0.872	0.877	0.875
		Validation	0.895	0.892	0.897	0.895
		Test	1	1	1	1
	Components = 10	Train	0.893	0.895	0.892	0.893
		Validation	0.895	0.897	0.892	0.895
		Test	0.889	0.887	0.892	0.889
	Components = 11	Train	0.911	0.91	0.912	0.911
		Validation	0.895	0.897	0.892	0.895
		Test	0.889	0.892	0.887	0.889
	Components = 12	Train	0.911	0.912	0.91	0.911
		Validation	0.947	0.945	0.95	0.947
		Test	0.889	0.887	0.892	0.889
	Components = 13	Train	0.946	0.945	0.947	0.946
		Validation	0.947	0.95	0.945	0.947
		Test	0.889	0.89	0.887	0.889
	Components = 14	Train	0.893	0.895	0.892	0.893
		Validation	0.895	0.897	0.892	0.895
		Test	0.944	0.945	0.942	0.944
	Components = 15	Train	0.929	0.93	0.928	0.929
		Validation	0.895	0.892	0.897	0.895
		Test	1	1	1	1
